# Correction: Academic global surgical competencies: A modified Delphi consensus study

**DOI:** 10.1371/journal.pgph.0002414

**Published:** 2023-09-14

**Authors:** Natalie Pawlak, Christine Dart, Hernan Sacoto Aguilar, Emmanuel Ameh, Abebe Bekele, Maria F. Jimenez, Kokila Lakhoo, Doruk Ozgediz, Nobhojit Roy, Girma Terfera, Adesoji O. Ademuyiwa, Barnabas Tobi Alayande, Nivaldo Alonso, Geoffrey A. Anderson, Stanley N. C. Anyanwu, Alazar Berhe Aregawi, Soham Bandyopadhyay, Tahmina Banu, Alemayehu Ginbo Bedada, Anteneh Gadisa Belachew, Fabio Botelho, Emmanuel Bua, Leticia Nunes Campos, Chris Dodgion, Michalina Drejza, Marcel E. Durieux, Rohini Dutta, Sarnai Erdene, Rodrigo Vaz Ferreira, Zipporah Gathuya, Dhruva Ghosh, Randeep Singh Jawa, Walter D. Johnson, Fauzia Anis Khan, Fanny Jamileth Navas Leon, Kristin L. Long, Jana B. A. Macleod, Anshul Mahajan, Rebecca G. Maine, Grace Zurielle C. Malolos, Craig D. McClain, Mary T. Nabukenya, Peter M. Nthumba, Benedict C. Nwomeh, Daniel Kinyuru Ojuka, Norgrove Penny, Martha A. Quiodettis, Jennifer Rickard, Lina Roa, Lucas Sousa Salgado, Lubna Samad, Justina Onyioza Seyi-Olajide, Martin Smith, Nichole Starr, Richard J. Stewart, John L. Tarpley, Julio L. Trostchansky, Ivan Trostchansky, Thomas G. Weiser, Adili Wobenjo, Elliot Wollner, Sudha Jayaraman

## Notice of republication

This article was republished on August 30, 2023, to correct missing content from Table 2. The publisher apologizes for the error.

In addition, the republished version of the article reflects the addition of an author, who was inadvertently excluded from the original publication. Corrections were also made to the author contributions.

Please download this article again to view the correct version. The originally published, uncorrected article and the republished, corrected articles are provided here for reference.

## Supporting information

S1 File Originally published, uncorrected article. (PDF)

S2 File Republished, corrected article. (PDF)

## References

[1] Pawlak N, Dart C, Aguilar HS, Ameh E, Bekele A, Jimenez MF, et al. (2023) Academic global surgical competencies: A modified Delphi consensus study. PLOS Glob Public Health 3(7): e0002102. 10.1371/journal.pgph.0002102 37450426 PMC10348592

