# Supplementary material for: Academic global surgical competencies: A modified Delphi consensus study
Source: PLOS Glob Public Health. 2023 Jul 14;3(7):e0002102. doi: 10.1371/journal.pgph.0002102 (PMC10348592; doi:10.1371/journal.pgph.0002102)
Supplement: Attachment — Submitted filename: PLOSGPH Jayaraman Response to Reviewers Letter final.docx [file pgph.0002102.s001.docx]

We thank the PLOS Global Health Editorial Office as well as the reviewers for their thoughtful review and feedback. Please see our responses to the points raised below. Additionally, Figure 3 has now been combined into Table 1, as suggested in your response. Thank you again for your continued consideration.

**Responses to Comments from Reviewer 1**

Abstract

Background, First sentence: I suggest the authors focus on academic global surgery rather than global surgery as a whole. Based on the same rationale and if the word limits allow it, I suggest adding "Academic" to the short title.

*We thank the reviewer for this comment. These changes have been made.*

Background, Third sentence: The purpose of the Delphi is not "to develop competencies" but rather to determine which competencies are essential. Please, edit the third sentence to reflect this.

*We thank the reviewer for this comment. These changes have been made.*

Results, First sentence: This sentence is a restatement of the first sentence in the methods. The only difference being the number of countries represented. Either focus on the methodology or criteria used to select the task force members in the methods section or use the results section sentence in place of the first sentence in the methods. Also, "Diverse" is a tautology here since diversity is exemplified by the number of countries represented.

*We thank the reviewer for this comment. The multi-step process of task force recruitment is too lengthy for the abstract, however, to make this sentence more focused on methodology, we have clarified that the broad group is identified through snowball sampling as the recruitment strategy.*

Conclusion, First sentence: It is more accurate to say the Delphi "identified" nine competencies rather than "led to."

*We thank the reviewer for this comment. The Conclusion has been edited to read, “This Delphi process, with input from experts worldwide, identified nine competencies which can be used to develop standardized academic global surgery and perioperative care curricula worldwide.”*

Manuscript

Introduction: It is perplexing that the authors do not define global surgery or academic global surgery in the first paragraph but use these terminologies only to state that there is no unified definition of global surgery. While this statement is true, it is essential that the authors state under which definitions of global surgery and academic global surgery they are working.

*We thank the reviewer for this comment. The reviewer has actually identified a key issue the authors try to highlight, which is that there is to date no universally accepted definition of academic global surgery or academic global surgery which we note in line 66. However, to address any confusion that might lead to the reader understanding that all global surgery practice might be academic, we move the discussion of the definition of global surgery to the Discussion section and added clarifications in the Introduction:*

- *In lines 71-73, we included* *the comment that competencies in Health Equity and Social Justice and Social and Environmental Determinants of Health are clearly important competencies for equitable and just health care delivery that are thus far missing in published global surgical curricula based on our previous systematic review.*
- *In Section titled Prioritizing the LMIC Context in Competency Development under the Discussion Section (Lines 261-295), we emphasize the concerns regarding definition of global surgery by stating: “Indeed, there is no consensus on what global surgery is nor how it should be taught in local, regional, or global contexts. To date, no standardized or universal competencies exist in academic global surgery and perioperative care.”*

The aforementioned lack of clarification has obscured the study's justification. In lines 79-80 the authors conclude that there is a need for standardized curricula on the fundamentals of academic global surgery based on a review of HIC training material. Are we therefore to understand that the aim of the study was the identification of competencies for the education of aspiring global surgeons in HICs?

*We thank the reviewer for this comment. This has been addressed in the previous edits. The systematic review of published global surgical curricula only found material from and by HICs which formed the basis for this project’s world-wide Delphi survey with explicit weighting of LMIC experts to create a* *universal, consensus-based framework for academic global surgery. We added a sentence about this gap in the Introduction, lines 78-80.*

Lines 82-94: This paragraph would probably be better placed in the discussion were it could expand on the importance of ethics and standardization.

*We thank the reviewer for this comment. These lines have been moved to paragraph two under “Prioritizing the LMIC Context in Curriculum Development” in the discussion (Lines 261-295).*

Line 118: Instead of "diverse" please elaborate on some of the criteria that were considered.

*We thank the reviewer for this comment. Lines 116-145 explain the criteria considered by the logistics team and Taskforce. An additional sentence has been added to further clarify the process: “Other criteria considered when selecting the expert panel included WHO region, country income level, surgical and perioperative specialty, and training level.” Further details regarding the composition of the expert panel can be seen in Table 1 and Figures 2-3.*

Lines 159-165: Please, define "core competency" and "sub-competency."

*We thank the reviewer for this comment. We have clarified this text further:* “*Competencies, and individual learning objectives comprising a competency, that did not meet the 80% threshold were removed from the framework.” Additional clarification is depicted in Table 2.*

Discussion: Please, put these competencies in context with regards to existing global surgery MSc/PhD programs, research fellowships, and other training program curricula.

*We thank the reviewer for this comment. The authors chose not to do so because the majority of the existing programs and fellowships are focused on HIC settings and we intentionally did not want to turn the gaze of this project to the HIC setting programs.*

**Responses to Comments from Reviewer 2**

A Delphi consensus is usually conducted in at least three rounds with the first round including qualitative open ended questions. Please comment why you chose to perform a modified Delphi consensus, provide references for your methodology, and describe much more clearly the process by which you created the first round instrument. In the modified Delphi technique the first round instrument is usually based on literature or preliminary research if it is structured, though it seems like this one was based on consensus of a smaller group of experts? How might that have influenced your findings? Could you comment specifically on how even the notion of global surgery may reflect a colonizing mindset? Is there any possibility that surgeons from LMICs may not even agree that there is or should be such a thing as academic global surgery and that the entire premise for the consensus is flawed? If you don't agree with this possibility that is fine, but I think it should be addressed. Again related to the methodology, how did you decide on two rounds and were there stopping criteria? Why the 5 point Likert scale?

*We thank the reviewer for this comment. We appreciate this question raised regarding the study methodology. Throughout qualitative study literature, the Delphi technique has evolved since its originally described approach that the reviewer mentions. According to a research methodology guide published in the BMJ (“What Are Delphi Studies?” by D. Barrlett and R. Heale), the common component of the Delphi technique is the use of consensus-driven rounds of voting through which experts provide their opinion on a given topic. Bartlett and Heale state, “Within this broad approach, there can be variation in areas such as how many rounds there are, how the questions are delivered and responses collected, and how ‘consensus’ is judged.” This reference and other published modified Delphi studies referenced in the manuscript (e.g., studies from the National Institute for Health Research Global Research Health Unit on Global Surgery and the Coronavirus Global Surgical Collaborative) validate modified approaches when performing an international consensus-based survey.*

*Additionally, edits have been made to the paragraph to explain why the first-round instrument was created (Lines 118-120) to provide clarification to the reviewer’s question regarding how the first-round instrument was created. The initial instrument was based on a thorough literature review (that is referenced in the manuscript), which is a validated approach to initiating the polling in a modified Delphi study, as the reviewer mentions.*

*In response to points made regarding whether the notion of global surgery reflects a colonizing mindset, the purpose of this multi-national study is to help define what academic global surgery entails across all geographic contexts (including the relationship between rural and urban settings, not just HIC-LMIC contexts). The notion of discussing decolonization as part of the global surgery education discourse was favored by several LMIC participants, which we discuss. However, developing consensus over the definition and perceptions of the notion of global surgery was beyond the scope of this project.*

*On a final note, we explicitly state that the most common definition of global surgery as a field carries the dangers of being in and of itself HIC-centric and that the definition fails to include the principles and content that are necessary for this field broadly with consistent input from LMIC experts.*

How did you determine the sample size? This is large for a Delphi study.

*We thank the reviewer for this comment. To prioritize inclusive and comprehensive consensus-focused polling on a global scale, no limitations were set for the sample size. The final sample size of n=59 is based on the responses after both rounds from the 76 experts who were initially reached about this project and is comparable to other recently published international Delphi studies, including research on the development of global surgery guidelines for the prevention of surgical‐site infection (n=57)^1^ or the safe return of surgeons to clinical practice in the Covid-19 pandemic (n=44).^2^*

*1) National Institute for Health Research Global Research Health Unit on Global Surgery. Delphi prioritization and development of global surgery guidelines for the prevention of surgical-site infection. Br J Surg. 2020;107(8):970-977. doi:10.1002/bjs.11530*

*2) Asbun HJ, Abu Hilal M, Kunzler F, et al. International Delphi Expert Consensus on Safe Return to Surgical and Endoscopic Practice: From the Coronavirus Global Surgical Collaborative. Ann Surg. 2021;274(1):50-56. doi:10.1097/SLA.0000000000004674*

While conceptually, I think this research adds value to our general understanding of what competencies should be in academic global surgery (and maybe even more so about what a group of experts think academic global surgery actually is), I'm struck by how most of these competencies apply more generally to academic activities in global health. I think it would be helpful if the authors could provide a discussion of the competencies identified by the Delphia [sic] panel related to competencies more generally in global health. Do those exist? Is there consensus there? How are these similar or different? What is the value for having distinct competencies in global surgery?

*We thank the reviewer for this comment. The reviewer highlights an important consideration of how academic global surgery differs from and compared to global health education. The Discussion section of the revised manuscript now includes mention of the Consortium of Universities for Global Health (CUGH) competencies as the current standard, as well as the weaknesses of this framework such as inadequate LMIC expert input. A new section has also been added to the Discussion titled, “Creating Competencies Specific to Academic Global Surgery” which compares the competencies specific to academic global surgery compared to those seen in graduate medical education for surgical trainees and competencies for global health leaders put forth by the Consortium of Universities for Global Health (Lines 307-311) and further elaborates on the removal of clinical practice competency which was noted in the Results section.*

It strikes me as especially noteworthy that competencies regarding clinical practice were removed during the consensus process. This deserves more emphasis, especially in the larger debate around what academic global surgery is and its vision, etc. Are you saying there is minimal or no role for clinical care and clinical training in academic global surgery? That is certainly a major departure from US-based global surgery practice.

*We thank the reviewer for this comment. This result is mentioned in the Results as well as in the first paragraph of the Discussion. This point is now also addressed in the new section described in the response above, in which we discuss how and why the decreased focused on clinical skills distinguishes academic global surgery competencies from graduate medical education competencies.*

A significant volume of surgery is provided by non-surgeons in LMICs. Did you think of including their perspectives? Why/why not? Is this a limitation?

*We thank the reviewer for this comment. While non-surgeons may contribute to clinical surgical care delivery, they have minimal roles in academic surgical training and therefore were not included in this process. Since this is the first attempt to develop consensus-based competencies, we certainly hope that their voices are included over time as this framework is used and revised in the future.*

Abstract-Background, "inform in" appears to be a typo.

*We thank the reviewer for this comment. This change has been made.*

Line 54, sentence starting in 2015 needs to be reworked.

*We thank the reviewer for this comment. This change has been made.*

Same for the First sentence in the Methods, it is very confusing.

*We thank the reviewer for this comment. This change has been made.*
